# Supplementary material for: The human microbiome and COVID-19: A systematic review
Source: PLoS One. 2021 Jun 23;16(6):e0253293. doi: 10.1371/journal.pone.0253293 (PMC8221462; doi:10.1371/journal.pone.0253293)
Supplement: S1 File — (DOCX) [file pone.0253293.s001.docx]

**S1 File**

The results of database search (Last accessed: 2020/Oct/31)

PubMed (<https://pubmed.ncbi.nlm.nih.gov>): Free word search, Additional filters, SPECIES “Humans”.

Search Terms: (“severe acute respiratory syndrome coronavirus 2” OR “SARS-CoV-2” OR “SARS2” OR “Wuhan coronavirus” OR “coronavirus” OR “COVID-19” OR “novel coronavirus” OR “COVID19” OR “nCoV” OR “coronavirus disease 2019”) AND (“microbiota*” OR “microbiome*” OR “mycobiome” OR “virome” OR “flora”).

Results: 127 articles.

Embase (<https://www.embase.com/#search>): Advanced search, Filters “Human”, Deduplication functions,

Search Terms: (“severe acute respiratory syndrome coronavirus 2” OR “SARS-CoV-2” OR “SARS2” OR “Wuhan coronavirus” OR “coronavirus” OR “COVID-19” OR “novel coronavirus” OR “COVID19” OR “nCoV” OR “coronavirus disease 2019”) AND (“microbiota*” OR “microbiome*” OR “mycobiome” OR “virome” OR “flora”).

Results: 83 articles.

Web of Science ([http://www.webofknowledge.com/wos](http://webofknowledge.com/WOS)): Web of science/core collection, All fields,

Search Terms: (“severe acute respiratory syndrome coronavirus 2” OR “SARS-CoV-2” OR “SARS2” OR “Wuhan coronavirus” OR “coronavirus” OR “COVID-19” OR “novel coronavirus” OR “COVID19” OR “nCoV” OR “coronavirus disease 2019”) AND (“microbiota*” OR “microbiome*” OR “mycobiome” OR “virome” OR “flora”).

Results: 296 articles

Cochrane Database of Systematic Reviews (<https://www.cochranelibrary.com>) Title Abstract Keyword search, Search Terms: (“severe acute respiratory syndrome coronavirus 2” OR “SARS-CoV-2” OR “SARS2” OR “Wuhan coronavirus” OR “coronavirus” OR “COVID-19” OR “novel coronavirus” OR “COVID19” OR “nCoV” OR “coronavirus disease 2019”) AND (“microbiota*” OR “microbiome*” OR “mycobiome” OR “virome” OR “flora”).

Results: 0 articles.

CINAHL Free word search, Search Terms: (“severe acute respiratory syndrome coronavirus 2” OR “SARS-CoV-2” OR “SARS2” OR “Wuhan coronavirus” OR “coronavirus” OR “COVID-19” OR “novel coronavirus” OR “COVID19” OR “nCoV” OR “coronavirus disease 2019”) AND (“microbiota*” OR “microbiome*” OR “mycobiome” OR “virome” OR “flora”).

Results: 37 articles.
