# Supplementary material for: The human microbiome and COVID-19: A systematic review
Source: PLoS One. 2021 Jun 23;16(6):e0253293. doi: 10.1371/journal.pone.0253293 (PMC8221462; doi:10.1371/journal.pone.0253293)
Supplement: S2 File — (DOCX) [file pone.0253293.s002.docx]

**S2 File. The classification criteria of COVID-19 severity.**

Reference No11: The criteria for clinical manifestation are as described in the previous study (Jian Wu, Jun Liu, Xinguo Zhao, Chengyuan Liu, Wei Wang, et al. Clinical Characteristics of Imported Cases of Coronavirus Disease 2019 (COVID-19) in Jiangsu Province: A Multicenter Descriptive Study, Clinical Infectious Diseases; 71(15) 706-712: 2020. <https://doi.org/10.1093/cid/ciaa199> ).

Reference No 14: National Health Commission of the People’s Republic of China, National Administration of Traditional Chinese Medicine. Guideline for diagnosis and treatment of COVID-19 (7th version).
