# Supplementary material for: The human microbiome and COVID-19: A systematic review
Source: PLoS One. 2021 Jun 23;16(6):e0253293. doi: 10.1371/journal.pone.0253293 (PMC8221462; doi:10.1371/journal.pone.0253293)
Supplement: S1 Table — (DOCX) [file pone.0253293.s003.docx]

**S1 Table:** Risk of bias for each study

| Risk of Bias | Selection of participants | Confounding variables | Measurement of exposure | Blinding of outcome assessment | Incomplete outcome data | Selective outcome reporting |
| --- | --- | --- | --- | --- | --- | --- |
| Zuo et al. (a) (2020) | Low | Unclear | Low | Low | Low | Unclear |
| Zuo et al. (b) (2020) | Low | Unclear | Low | Low | Low | Unclear |
| Zuo et al. (c) (2020) | Unclear | Unclear | Low | Low | Low | Unclear |
| De Maio et al. (2020) | Low | Unclear | Low | Low | Low | Unclear |
| Gu et al. (2020) | Low | Unclear | Low | Low | Low | Unclear |
| Shen et al. (2020) | Low | Unclear | Low | Low | Low | Unclear |
| Zhang et al. (2020) | Low | Unclear | Low | Low | Low | Unclear |
| Chen et al. (2020) | Unclear | Unclear | Low | Low | Low | Unclear |
| Fan et al. (2020) | Unclear | Unclear | Low | Low | Low | Unclear |
| Ren et al. (2020) | Unclear | Unclear | Low | Low | Unclear | Unclear |
| Tao et al. (2020) | Low | Unclear | Low | Low | Low | Unclear |
| Yu et al. (unpublished) | High | Unclear | Low | Low | Unclear | Unclear |
| Ai et al. (unpublished) | Low | Unclear | Low | Low | Low | Unclear |
| Budding et al. (unpublished) | Low | Unclear | Low | Low | Low | Unclear |
| Xu et al. (a) (abstract only) | Unclear | Unclear | Unclear | Low | Unclear | Unclear |
| Xu et al. (b) (abstract only) | Unclear | Unclear | Unclear | Low | Unclear | Unclear |
