# Supplementary material for: The human microbiome and COVID-19: A systematic review
Source: PLoS One. 2021 Jun 23;16(6):e0253293. doi: 10.1371/journal.pone.0253293 (PMC8221462; doi:10.1371/journal.pone.0253293)
Supplement: Attachment — Submitted filename: Rev. Microbiota and Covid-19.docx [file pone.0253293.s005.docx]

In this systematic review, the authors collected evidence on alterations in the gut, upper and lower respiratory microbiota in Covid-19 infection.

The study is well conducted and written, offering a good perspective on the work in literature.

The evidence collected is unfortunately of low grade. All the studies collected had an observational design, 14/16 are conducted in China and consider only hospitalized patients. For these reasons, many confounding factors may have altered the results. Moreover, different types of samples and different methods of analysis are used.

Major revision

- Clarify whether unpublished studies were reviewed by a third-party researcher not involved in the study.

Minor revision

- The design and characteristics of the included studies are well described in Table 1. However, they could be reported more clearly in the text.

- In the "Selection criteria and search strategy" paragraph, words used for the research could be placed in quotation marks rather than in parentheses.
